# Repeatability and reliability of choriocapillaris flow deficit measurement by spectral-domain OCTA

**DOI:** 10.1007/s00417-024-06423-y

**Published:** 2024-02-28

**Authors:** Melih Tarhan, Thomas Lehmann, Daniel Meller, Martin Hammer

**Affiliations:** 1 Department of Ophthalmology, University Hospital Jena, Am Klinikum 1, 07747 Jena, Germany; 2 Institute of Medical Statistics, Computer and Data Sciences, University Hospital Jena, Jena, Germany; 3 Center for Medical Optics and Photonics, Univ. of Jena, Jena, Germany

**Keywords:** OCT angiography, Choriocapillaris, OCTA


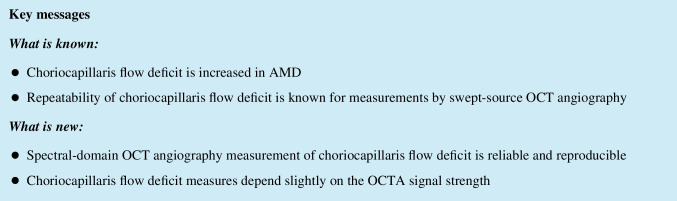


Choriocapillaris (cc) flow deficit (FD) is a new optical coherence tomography angiography (OCTA) measure to describe possible undersupply of the outer retina with oxygen and nutrients. In age-related macular degeneration (AMD), FD was shown to be associated with drusen [1] as well as the enlargement of geographic atrophy [2] and was higher in eyes having subretinal drusenoid deposits [3]. However, FD has to be determined from the OCTA data cube by mathematical methods, which may introduce systematic errors as well as noise to the measurement [4]. Thus, the repeatability and reliability of this measure are important for the interpretation of FD and its relation to clinical symptoms of the disease. Repeatability is investigated for swept-source (SS)-OCTA [5], mostly used in experimental studies, however, not for spectral-domain (SD)-OCTA, which is widely available for clinical routine investigations. Thus, here, we investigated the repeatability and reliability of SD-OCTA (Zeiss Cirrus 5000, Carl Zeiss Meditec Inc., Dublin, USA).

We did five repeated 6 × 6 mm macular OCTA scans in 13 healthy young subjects (mean age: 30.2 ± 7.7 years). The cc was automatically segmented as the slab 29 to 49 µm underneath the retinal pigment epithelium (RPE). The OCTA image, obtained from the optical microangiography algorithm utilizing amplitude and phase differences of sequential B-scans, was multiplied with the inverse structural OCT image of the slab in order to compensate for shadowing artifacts from structures anterior to the cc [4]. A Phansalkar local threshold with a radius of 4 pixels (26.4 µm in an emmetropic eye [4]) was applied to the compensated cc OCTA image, and the fraction of non-perfused pixels was determined as FD in the center (c), inner (ir), and outer ring (or) of an ETDRS grid centered to the fovea using the retinal OCTA image (Fig. 1).

**Fig. 1:**
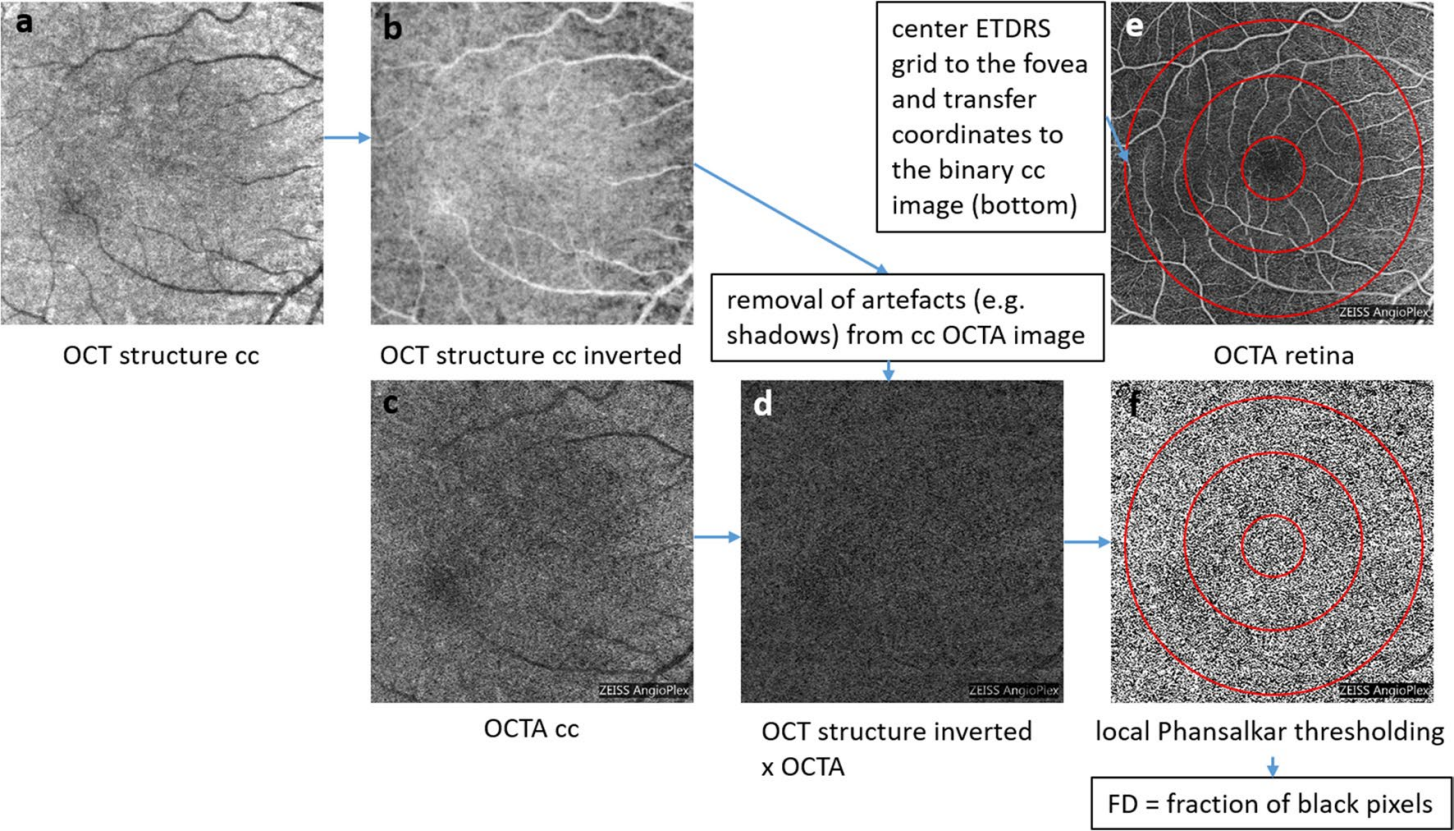
Workflow of choriocapillaris flow deficit calculation: The OCT structure image of the cc (**a**) is inverted (**b**) and multiplied with the OCTA image (**c**) to get an OCTA image compensated for artifacts (**d**). Local thresholding is applied to this image (**f**), and the fraction of black pixels in this image is determined as FD within the center, the inner, and the outer ring of the ETDRS grid, centered at the fovea in the OCTA image of the retina (**e**) and transferred to the thresholded cc OCTA image (**f**)

The mean FD values were 0.450 ± 0.042 (c), 0.435 ± 0.040 (ir), and 0.407 ± 0.046 (or). Repeatability is described by the mean within-case coefficient of variation over the five measures: 0.066 ± 0.037 (c), 0.061 ± 0.029 (ir), and 0.066 ± 0.035 (or). The reliability was measured by the intra-class correlation coefficient (ICC): 0.567 (c, 95%-CI 0.322–0.806), 0.620 (ir, 0.382–0.836), and 0.684 (or, 0.463–0.869). No age dependence of the FD was found (age range 21–45 years). In order to investigate the influence of the OCTA signal strength on FD, but eliminate inter-individual differences of FD, a relative FD was calculated as the ratio of the single measurement and the average per subject. This revealed a slight but significant (*p* = 0.011) increase of FD with decreasing signal strength (*R* =  − 0.182, Fig. 2). Besides signal strength, also, the thickness of Bruch’s membrane could have an influence on the measured FD values.

**Fig. 2:**
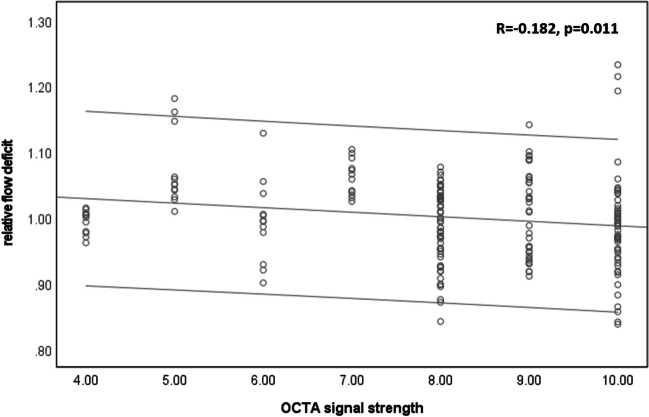
Plot of relative flow deficit vs. OCTA signal strength with best linear fit and 95% confidence interval indicating virtually higher FD at lower signal strength

Our investigations indicate moderate reliability for cc FD measurement using SD-OCTA. The repeatability, however, is high as shown by a within-case coefficient of variation of less than 7%. This vindicates the use of SD-OCTA for the assessment of cc in studies as well as routine clinical investigation. The absolute values of FD, however, differ between SD-OCTA and SS-OCTA. Whereas our values of 40–45% FD resemble that of other investigators using FD-OCTA [1], values reported from SS-OCTA are much lower [3–5]. The reason might be a higher resolution of SS-OCTA due to a higher A-scan rate. But also, a difference in the cc segmentation (16 to 31 µm beneath RPE [4, 5] or 4 to 20 µm underneath Bruch’s membrane [3] in SS-OCTA) can affect the measured FD.

In conclusion, SD-OCTA measurement of relative FD is regarded as a reliable and reproducible technique for cc blood flow deficit measurement.

## Declarations

This study adhered to the tenets of the Declaration of Helsinki and informed consent was given by the participants.

## References

[1] Nassisi M, Tepelus T, Nittala MG, Sadda SR (2019). Choriocapillaris flow impairment predicts the development and enlargement of drusen. Graefes Arch Clin Exp Ophthalmol.

[2] Alagorie AR, Nassisi M, Verma A, Nittala M, Corradetti G, Velaga S, Sadda SR (2020). Relationship between proximity of choriocapillaris flow deficits and enlargement rate of geographic atrophy. Graefes Arch Clin Exp Ophthalmol.

[3] Li J, Liu Z, Lu J, Shen M, Cheng Y, Siddiqui N, Zhou H, Zhang Q, Liu J, Herrera G, Hiya FE, Gregori G, Wang RK, Rosenfeld PJ (2023). Decreased macular choriocapillaris perfusion in eyes with macular reticular pseudodrusen imaged with swept-source OCT angiography. Invest Ophthalmol Vis Sci.

[4] Chu Z, Cheng Y, Zhang Q, Zhou H, Dai Y, Shi Y, Gregori G, Rosenfeld PJ, Wang RK (2020). Quantification of choriocapillaris with Phansalkar local thresholding: pitfalls to avoid. Am J Ophthalmol.

[5] Zhang Q, Zheng F, Motulsky EH, Gregori G, Chu Z, Chen CL, Li C, de Sisternes L, Durbin M, Rosenfeld PJ, Wang RK (2018). A novel strategy for quantifying choriocapillaris flow voids using swept-source OCT angiography. Invest Ophthalmol Vis Sci.

